# (*E*)-1-Ferrocenyl-3-(2-fur­yl)prop-2-en-1-one

**DOI:** 10.1107/S1600536810011001

**Published:** 2010-03-31

**Authors:** Yong-Hong Liu, Rong Guo

**Affiliations:** a College of Chemistry and Chemical Engineering, Yangzhou University, Yangzhou 225002, People’s Republic of China

## Abstract

The title compound, [Fe(C_5_H_5_)(C_12_H_9_O_2_)], exhibits an *E* configuration. In the ferrocene unit, the two cyclo­penta­dienyl rings are almost parallel [dihedral angle = 0.76 (12)°] and the C atoms are in an eclipsed conformation. An intra­molecular C—H⋯O hydrogen bond generates an *S(5)* ring. In the crystal, the mol­ecules are linking into zigzag chains *via* two C—H⋯O hydrogen-bonding inter­actions along the *c* axis and neighbouring chains are stabilized by electrostatic inter­action forces.

## Related literature

For the biological activity of chalcones and chalcone derivatives, see: Liu *et al.* (2003 ▶). For the ability of some chalcones to block voltage-dependent potassium channels, see: Yarishkin *et al.* (2008 ▶). Replacement of the aromatic group of penicillins and cephalosporins by a ferrocenyl group could improve their anti­biotic activity, see: Edwards *et al.* (1975 ▶). For our ongoing research in this area, see: Shi *et al.* (2004 ▶); Liu, Liu *et al.* (2008 ▶). For the synthesis, see: Huang *et al.* (1998 ▶). For a related structure, see: Liu, Ye *et al.* (2008 ▶) For graph-set notations of ring systems, see: Bernstein *et al.* (1995 ▶). For related literature, see: Zhai *et al.* (1999 ▶).
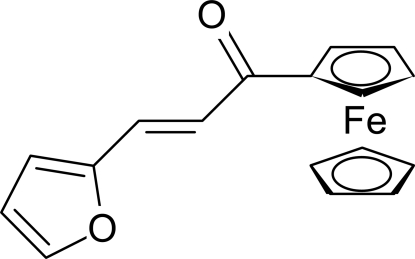


## Experimental

### 

#### Crystal data

[Fe(C_5_H_5_)(C_12_H_9_O_2_)]
*M* *_r_* = 306.13
Orthorhombic, 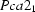

*a* = 9.0677 (13) Å
*b* = 14.222 (2) Å
*c* = 10.4846 (15) Å
*V* = 1352.1 (3) Å^3^
*Z* = 4
Mo *K*α radiation
μ = 1.11 mm^−1^
*T* = 296 K
0.28 × 0.25 × 0.22 mm

#### Data collection

Bruker SMART 1000 CCD diffractometer
Absorption correction: multi-scan (*SADABS*; Bruker, 2002 ▶) *T*_min_ = 0.746, *T*_max_ = 0.792
11058 measured reflections
3012 independent reflections
2772 reflections with *I* > 2σ(*I*)
*R*_int_ = 0.035

#### Refinement

*R*[*F* ^2^ > 2σ(*F* ^2^)] = 0.023
*wR*(*F* ^2^) = 0.058
*S* = 1.00
3012 reflections
182 parameters
1 restraint
H-atom parameters constrained
Δρ_max_ = 0.20 e Å^−3^
Δρ_min_ = −0.19 e Å^−3^
Absolute structure: Flack (1983 ▶), 1340 Friedel pairs
Flack parameter: 0.012 (14)

### 

Data collection: *SMART* (Bruker, 2002 ▶); cell refinement: *SAINT* (Bruker, 2002 ▶); data reduction: *SAINT*; program(s) used to solve structure: *SHELXS97* (Sheldrick, 2008 ▶); program(s) used to refine structure: *SHELXL97* (Sheldrick, 2008 ▶); molecular graphics: *PLATON* (Spek, 2009 ▶); software used to prepare material for publication: *SHELXTL* (Sheldrick, 2008 ▶).

## Supplementary Material

Crystal structure: contains datablocks I, global. DOI: 10.1107/S1600536810011001/pv2265sup1.cif

Structure factors: contains datablocks I. DOI: 10.1107/S1600536810011001/pv2265Isup2.hkl

Additional supplementary materials:  crystallographic information; 3D view; checkCIF report

## Figures and Tables

**Table 1:** Hydrogen-bond geometry (Å, °)

*D*—H⋯*A*	*D*—H	H⋯*A*	*D*⋯*A*	*D*—H⋯*A*
C13—H13⋯O1	0.93	2.45	2.797 (3)	102
C6—H6⋯O1^i^	0.93	2.56	3.473 (3)	166
C12—H12⋯O1^i^	0.93	2.71	3.576 (4)	155

Symmetry code: (i) 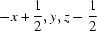.

**Fig. 1.**
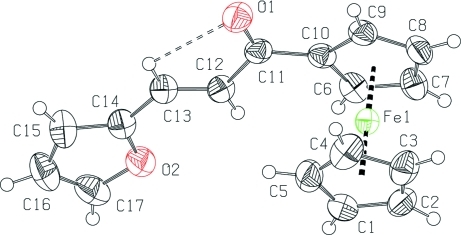
The molecular structure of the title compound, showing 50% probability ellipsoids. The C–H···O intra-molecular hydrogen bond is shown as dashed lines.

**Fig. 2.**
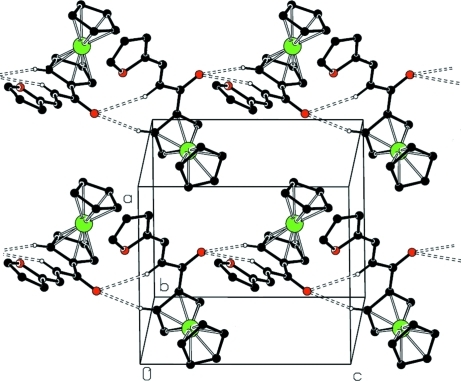
Unit cell packing of the title compound, showing the inter-molecular hydrogen bonds C–H···O as dashed lines. For the sake of clarity, H atoms not involved in hydrogen bonding have been omitted.

## supplementary crystallographic information

### Comment

Chalcone and its derivatives, as a natural produce, have shown strong antibacterial, antifungal, antitumor and anti-inflammatory properties (Liu *et al.*, 2003). Some chalcones demonstrated the ability to block voltage-dependent potassium channels (Yarishkin *et al.*, 2008). It has been demonstrated that the replacement of the aromatic group by the ferrocenyl moiety in penicillins and cephalosporins could improve their antibiotic activity (Edwards *et al.*, 1975). As on going research (Liu & Liu *et al.*, 2008; Shi *et al.*, 2004), we report herein the structure of the title compound.

The molecule of the title compound exists in the most stable configuration of (*E*)-isomer (Fig. 1). All of the C and O atoms are *sp*^2^-hybrid resulting in two large conjugated systems: one is formed by C1-C5 atoms and the other by the rest of the atoms. There is an intra-molecular hydrogen-bond C13–H13···O1 resulting in a five membered ring, *S(5)* in graph set notation (Bernstein *et al.*, 1995). The atoms O1/C11/C12/C13 are essentially planar and their mean-plane lies at 3.10 (14) and 16.35 (13) °, respectively, with the mean-planes of the furyl ring and the substituted cyclopentadienyl ring. In the ferrocene moiety, the Cps plane and Cp (the unsubstituted cyclopentadienyl ring) plane are almost parallel and the C atoms of Cp and Cps are in the eclipsed conformation. The Fe atom is slightly near the Cps palne as the distances Fe–Cgs and Fe–Cg are 1.6464 (9) and 1.6574 (10) Å, respectively, where Cgs and Cg are the centroids of Cps and Cp, respectively. The Cgs—Fe—Cg angle is 179.02 (5)°. The molecular dimensions agree very well with the corresponding dimensions reparted for the crystal structure of a similar compound (Liu & Ye *et al.*, 2008).

In the crystal structure, inter-molecular hydrogen-bonds of the type C—H···O, along the c axis, generate a *R*_2_^1^(7) motif (Bernstein *et al.*, 1995), linking the adjacent molecules into a zig-zag chain (Fig. 2, Tab. 1). Further more, the chain and its neighboring inverse parallel chains are stablilized by electrostatic interaction forces.

### Experimental

The title compound was synthesized according to the literature procedure (Huang *et al.*, 1998). An aqueous solution of potassium hydroxide (5%, 5 ml) was added slowly with stirring to a mixture of 2-furanylaldehyde (4.0 g, 0.043 mol) and acetoylferrocene (0.98 g, 0.043 mol) in ethanol (20 ml) in ice bath. The resulting mixture was stirred at room temperature for 4 h. The dark-red precipitated solid was filtered off, washed with water, dried and recrystallized from 95% ethanol (yield, 83%; M.P. 429.5-430.8 K. Crystals of (I) suitable for X-ray diffraction were obtained by slow evaporation of a solution of the solid in dichloromethane/ ether (4:1 v/v) at room temperature over a period of 6 d.

### Refinement

After their location in a difference map, all H atoms were fixed geometrically at ideal positions and allowed to ride on the parent C atoms, with C—H = 0.93 Å and *U_iso_*(H) = *1.2U_eq_*(C). An absolute structure was determined using anomalous dispersion effects employing 1353 Friedel pairs which were not merged.

### Crystal data

**Table:**

[Fe(C_5_H_5_)(C_12_H_9_O_2_)]	*D*_x_ = 1.504 Mg m^−^^3^
*M**_r_* = 306.13	Melting point: 429.5 K
Orthorhombic, *P**c**a*2_1_	Mo *K*α radiation, λ = 0.71073 Å
Hall symbol: P 2c -2ac	Cell parameters from 6056 reflections
*a* = 9.0677 (13) Å	θ = 2.7–27.4°
*b* = 14.222 (2) Å	µ = 1.11 mm^−^^1^
*c* = 10.4846 (15) Å	*T* = 296 K
*V* = 1352.1 (3) Å^3^	Prism, orange
*Z* = 4	0.28 × 0.25 × 0.22 mm
*F*(000) = 632	

### Data collection

**Table:**

Bruker SMART 1000 CCD diffractometer	3012 independent reflections
Radiation source: fine-focus sealed tube	2772 reflections with *I* > 2σ(*I*)
graphite	*R*_int_ = 0.035
φ and ω scans	θ_max_ = 27.7°, θ_min_ = 2.7°
Absorption correction: multi-scan (*SADABS*; Bruker, 2002)	*h* = −11→11
*T*_min_ = 0.746, *T*_max_ = 0.792	*k* = −18→16
11058 measured reflections	*l* = −13→13

### Refinement

**Table:**

Refinement on *F*^2^	Hydrogen site location: inferred from neighbouring sites
Least-squares matrix: full	H-atom parameters constrained
*R*[*F*^2^ > 2σ(*F*^2^)] = 0.023	*w* = 1/[σ^2^(*F*_o_^2^) + (0.0253*P*)^2^ + 0.1177*P*] where *P* = (*F*_o_^2^ + 2*F*_c_^2^)/3
*wR*(*F*^2^) = 0.058	(Δ/σ)_max_ = 0.001
*S* = 1.00	Δρ_max_ = 0.20 e Å^−^^3^
3012 reflections	Δρ_min_ = −0.18 e Å^−^^3^
182 parameters	Extinction correction: *SHELXL97* (Sheldrick, 2008), Fc^*^=kFc[1+0.001xFc^2^λ^3^/sin(2θ)]^-1/4^
1 restraint	Extinction coefficient: 0.0184 (11)
Primary atom site location: structure-invariant direct methods	Absolute structure: Flack (1983), 1340 Friedel pairs
Secondary atom site location: difference Fourier map	Flack parameter: 0.012 (14)

### Special details

**Table:**

Experimental. Analysis found (calculated) for C_17_H_14_FeO_2_ (%): C 66.61 (66.70), H 4.56 (4.61).
Geometry. All esds (except the esd in the dihedral angle between two l.s. planes) are estimated using the full covariance matrix. The cell esds are taken into account individually in the estimation of esds in distances, angles and torsion angles; correlations between esds in cell parameters are only used when they are defined by crystal symmetry. An approximate (isotropic) treatment of cell esds is used for estimating esds involving l.s. planes.
Refinement. Refinement of *F*^2^ against ALL reflections. The weighted *R*-factor wR and goodness of fit *S* are based on *F*^2^, conventional *R*-factors *R* are based on *F*, with *F* set to zero for negative *F*^2^. The threshold expression of *F*^2^ > σ(*F*^2^) is used only for calculating *R*-factors(gt) etc. and is not relevant to the choice of reflections for refinement. *R*-factors based on *F*^2^ are statistically about twice as large as those based on *F*, and *R*- factors based on ALL data will be even larger.

### Fractional atomic coordinates and isotropic or equivalent isotropic displacement parameters (Å^2^)

**Table:**

	*x*	*y*	*z*	*U*_iso_*/*U*_eq_	
Fe1	−0.04553 (2)	0.652980 (14)	0.19388 (4)	0.03343 (8)	
O1	0.35975 (15)	0.71855 (11)	0.24502 (15)	0.0524 (4)	
O2	0.28635 (15)	0.93776 (10)	−0.13390 (15)	0.0525 (3)	
C1	−0.1739 (2)	0.76594 (14)	0.1431 (2)	0.0523 (5)	
H1	−0.1789	0.7934	0.0627	0.063*	
C2	−0.26341 (18)	0.69146 (14)	0.1893 (3)	0.0522 (4)	
H2	−0.3381	0.6615	0.1440	0.063*	
C3	−0.2206 (2)	0.67061 (16)	0.3142 (2)	0.0550 (6)	
H3	−0.2613	0.6243	0.3661	0.066*	
C4	−0.1041 (2)	0.73260 (17)	0.3481 (2)	0.0582 (6)	
H4	−0.0554	0.7344	0.4261	0.070*	
C5	−0.0756 (2)	0.79058 (15)	0.2428 (3)	0.0555 (6)	
H5	−0.0042	0.8374	0.2390	0.067*	
C6	0.0731 (2)	0.61272 (14)	0.03780 (19)	0.0430 (4)	
H6	0.0722	0.6417	−0.0417	0.052*	
C7	−0.0200 (2)	0.53753 (14)	0.0779 (2)	0.0484 (5)	
H7	−0.0924	0.5087	0.0287	0.058*	
C8	0.0167 (2)	0.51418 (13)	0.2055 (3)	0.0493 (5)	
H8	−0.0278	0.4675	0.2543	0.059*	
C9	0.13284 (19)	0.57416 (13)	0.2463 (2)	0.0455 (4)	
H9	0.1776	0.5736	0.3261	0.055*	
C10	0.16853 (19)	0.63550 (13)	0.14257 (19)	0.0385 (4)	
C11	0.27507 (18)	0.71373 (13)	0.15362 (18)	0.0386 (4)	
C12	0.2747 (2)	0.78616 (13)	0.05229 (18)	0.0398 (4)	
H12	0.2132	0.7793	−0.0181	0.048*	
C13	0.3620 (2)	0.86111 (13)	0.06132 (19)	0.0418 (4)	
H13	0.4231	0.8638	0.1325	0.050*	
C14	0.3723 (2)	0.93831 (14)	−0.02664 (19)	0.0436 (4)	
C15	0.4527 (2)	1.01887 (16)	−0.0245 (2)	0.0568 (6)	
H15	0.5206	1.0363	0.0376	0.068*	
C17	0.3148 (3)	1.02013 (17)	−0.1960 (3)	0.0614 (6)	
H16	0.2704	1.0383	−0.2720	0.074*	
C16	0.4146 (3)	1.07107 (16)	−0.1333 (3)	0.0630 (6)	
H17	0.4516	1.1295	−0.1569	0.076*	

### Atomic displacement parameters (Å^2^)

**Table:**

	*U*^11^	*U*^22^	*U*^33^	*U*^12^	*U*^13^	*U*^23^
Fe1	0.03501 (11)	0.03456 (13)	0.03073 (12)	−0.00320 (8)	0.00069 (13)	−0.00016 (13)
O1	0.0483 (7)	0.0608 (9)	0.0481 (8)	−0.0051 (6)	−0.0080 (6)	0.0125 (7)
O2	0.0544 (8)	0.0487 (8)	0.0545 (9)	−0.0015 (6)	−0.0052 (7)	0.0061 (7)
C1	0.0495 (11)	0.0416 (11)	0.0659 (14)	0.0076 (8)	−0.0004 (9)	0.0000 (9)
C2	0.0351 (7)	0.0549 (10)	0.0666 (13)	−0.0006 (7)	−0.0031 (11)	−0.0056 (15)
C3	0.0449 (10)	0.0650 (14)	0.0551 (14)	−0.0073 (9)	0.0148 (9)	−0.0063 (11)
C4	0.0514 (11)	0.0724 (15)	0.0507 (13)	−0.0033 (10)	0.0060 (9)	−0.0240 (12)
C5	0.0476 (10)	0.0414 (11)	0.0775 (17)	−0.0027 (8)	0.0095 (10)	−0.0170 (10)
C6	0.0554 (10)	0.0400 (10)	0.0336 (10)	0.0012 (8)	0.0074 (8)	−0.0022 (8)
C7	0.0596 (11)	0.0368 (10)	0.0487 (12)	−0.0034 (9)	0.0028 (9)	−0.0078 (9)
C8	0.0535 (9)	0.0335 (8)	0.0611 (14)	−0.0005 (7)	0.0061 (12)	0.0097 (12)
C9	0.0433 (9)	0.0439 (11)	0.0493 (11)	0.0046 (8)	0.0006 (8)	0.0142 (8)
C10	0.0380 (8)	0.0394 (9)	0.0380 (9)	0.0069 (7)	0.0059 (7)	0.0036 (7)
C11	0.0328 (8)	0.0436 (10)	0.0394 (10)	0.0044 (7)	0.0058 (7)	0.0046 (7)
C12	0.0391 (9)	0.0454 (10)	0.0348 (10)	−0.0003 (8)	0.0023 (7)	0.0036 (8)
C13	0.0436 (9)	0.0451 (10)	0.0365 (10)	−0.0010 (8)	0.0045 (7)	0.0005 (8)
C14	0.0460 (10)	0.0441 (11)	0.0408 (10)	−0.0008 (8)	0.0072 (8)	−0.0030 (8)
C15	0.0697 (14)	0.0522 (13)	0.0486 (13)	−0.0186 (10)	0.0019 (9)	−0.0009 (10)
C17	0.0744 (14)	0.0521 (13)	0.0578 (15)	0.0124 (11)	0.0043 (12)	0.0136 (10)
C16	0.0848 (16)	0.0428 (12)	0.0614 (15)	−0.0070 (12)	0.0217 (13)	0.0049 (11)

### Geometric parameters (Å, °)

**Table:**

Fe1—C10	2.0295 (18)	C5—H5	0.9300
Fe1—C6	2.0404 (19)	C6—C7	1.426 (3)
Fe1—C5	2.041 (2)	C6—C10	1.435 (3)
Fe1—C9	2.0431 (18)	C6—H6	0.9300
Fe1—C3	2.044 (2)	C7—C8	1.418 (4)
Fe1—C4	2.044 (2)	C7—H7	0.9300
Fe1—C2	2.0506 (17)	C8—C9	1.421 (3)
Fe1—C1	2.054 (2)	C8—H8	0.9300
Fe1—C7	2.056 (2)	C9—C10	1.431 (3)
Fe1—C8	2.0566 (18)	C9—H9	0.9300
O1—C11	1.230 (2)	C10—C11	1.478 (3)
O2—C17	1.365 (3)	C11—C12	1.480 (3)
O2—C14	1.369 (2)	C12—C13	1.331 (3)
C1—C5	1.418 (3)	C12—H12	0.9300
C1—C2	1.419 (3)	C13—C14	1.437 (3)
C1—H1	0.9300	C13—H13	0.9300
C2—C3	1.398 (4)	C14—C15	1.358 (3)
C2—H2	0.9300	C15—C16	1.404 (4)
C3—C4	1.421 (3)	C15—H15	0.9300
C3—H3	0.9300	C17—C16	1.332 (4)
C4—C5	1.402 (4)	C17—H16	0.9300
C4—H4	0.9300	C16—H17	0.9300
			
C10—Fe1—C6	41.30 (8)	C5—C4—C3	107.8 (2)
C10—Fe1—C5	108.18 (8)	C5—C4—Fe1	69.82 (13)
C6—Fe1—C5	122.75 (9)	C3—C4—Fe1	69.63 (12)
C10—Fe1—C9	41.14 (7)	C5—C4—H4	126.1
C6—Fe1—C9	69.16 (9)	C3—C4—H4	126.1
C5—Fe1—C9	124.36 (9)	Fe1—C4—H4	126.0
C10—Fe1—C3	157.20 (9)	C4—C5—C1	108.63 (19)
C6—Fe1—C3	159.93 (9)	C4—C5—Fe1	70.04 (12)
C5—Fe1—C3	67.88 (9)	C1—C5—Fe1	70.24 (12)
C9—Fe1—C3	121.09 (9)	C4—C5—H5	125.7
C10—Fe1—C4	121.74 (9)	C1—C5—H5	125.7
C6—Fe1—C4	157.94 (9)	Fe1—C5—H5	125.6
C5—Fe1—C4	40.14 (11)	C7—C6—C10	107.50 (18)
C9—Fe1—C4	107.27 (10)	C7—C6—Fe1	70.23 (11)
C3—Fe1—C4	40.70 (9)	C10—C6—Fe1	68.95 (11)
C10—Fe1—C2	161.43 (10)	C7—C6—H6	126.3
C6—Fe1—C2	124.34 (11)	C10—C6—H6	126.3
C5—Fe1—C2	67.74 (8)	Fe1—C6—H6	126.1
C9—Fe1—C2	156.26 (9)	C8—C7—C6	108.35 (18)
C3—Fe1—C2	39.92 (11)	C8—C7—Fe1	69.85 (12)
C4—Fe1—C2	67.69 (10)	C6—C7—Fe1	69.03 (11)
C10—Fe1—C1	124.69 (8)	C8—C7—H7	125.8
C6—Fe1—C1	108.09 (9)	C6—C7—H7	125.8
C5—Fe1—C1	40.50 (9)	Fe1—C7—H7	126.9
C9—Fe1—C1	161.34 (8)	C7—C8—C9	108.49 (18)
C3—Fe1—C1	67.89 (10)	C7—C8—Fe1	69.82 (11)
C4—Fe1—C1	67.95 (10)	C9—C8—Fe1	69.21 (10)
C2—Fe1—C1	40.46 (8)	C7—C8—H8	125.8
C10—Fe1—C7	68.76 (8)	C9—C8—H8	125.8
C6—Fe1—C7	40.74 (8)	Fe1—C8—H8	126.8
C5—Fe1—C7	158.29 (10)	C8—C9—C10	107.7 (2)
C9—Fe1—C7	68.39 (9)	C8—C9—Fe1	70.23 (10)
C3—Fe1—C7	123.40 (9)	C10—C9—Fe1	68.92 (10)
C4—Fe1—C7	159.94 (10)	C8—C9—H9	126.1
C2—Fe1—C7	107.92 (9)	C10—C9—H9	126.1
C1—Fe1—C7	122.34 (9)	Fe1—C9—H9	126.3
C10—Fe1—C8	68.64 (8)	C9—C10—C6	107.91 (16)
C6—Fe1—C8	68.50 (10)	C9—C10—C11	123.17 (18)
C5—Fe1—C8	160.39 (11)	C6—C10—C11	128.60 (17)
C9—Fe1—C8	40.57 (8)	C9—C10—Fe1	69.94 (10)
C3—Fe1—C8	107.12 (10)	C6—C10—Fe1	69.76 (10)
C4—Fe1—C8	123.79 (12)	C11—C10—Fe1	120.80 (13)
C2—Fe1—C8	121.45 (8)	O1—C11—C10	120.74 (17)
C1—Fe1—C8	157.13 (10)	O1—C11—C12	121.46 (17)
C7—Fe1—C8	40.33 (11)	C10—C11—C12	117.79 (16)
C17—O2—C14	106.22 (17)	C13—C12—C11	120.34 (18)
C5—C1—C2	107.0 (2)	C13—C12—H12	119.8
C5—C1—Fe1	69.26 (12)	C11—C12—H12	119.8
C2—C1—Fe1	69.63 (11)	C12—C13—C14	127.24 (19)
C5—C1—H1	126.5	C12—C13—H13	116.4
C2—C1—H1	126.5	C14—C13—H13	116.4
Fe1—C1—H1	126.2	C15—C14—O2	108.90 (19)
C3—C2—C1	108.6 (2)	C15—C14—C13	132.0 (2)
C3—C2—Fe1	69.77 (11)	O2—C14—C13	119.06 (17)
C1—C2—Fe1	69.91 (10)	C14—C15—C16	107.5 (2)
C3—C2—H2	125.7	C14—C15—H15	126.3
C1—C2—H2	125.7	C16—C15—H15	126.3
Fe1—C2—H2	126.2	C16—C17—O2	111.1 (2)
C2—C3—C4	108.0 (2)	C16—C17—H16	124.4
C2—C3—Fe1	70.31 (12)	O2—C17—H16	124.4
C4—C3—Fe1	69.67 (12)	C17—C16—C15	106.3 (2)
C2—C3—H3	126.0	C17—C16—H17	126.9
C4—C3—H3	126.0	C15—C16—H17	126.9
Fe1—C3—H3	125.6		

### Hydrogen-bond geometry (Å, °)

**Table:**

*D*—H···*A*	*D*—H	H···*A*	*D*···*A*	*D*—H···*A*
C13—H13···O1	0.93	2.45	2.797 (3)	102
C6—H6···O1^i^	0.93	2.56	3.473 (3)	166
C12—H12···O1^i^	0.93	2.71	3.576 (4)	155

Symmetry codes: (i) −*x*+1/2, *y*, *z*−1/2.
